# The value of different imaging methods in the diagnosis of breast cancer: A protocol for network meta-analysis of diagnostic test accuracy

**DOI:** 10.1097/MD.0000000000025803

**Published:** 2021-05-14

**Authors:** Mei Zhang, Rongna Lian, Ruinian Zhang, Yulong Hong, Wen Feng, Shifang Feng

**Affiliations:** a Department of Radiology, Gansu Cancer Hospital; b The First Clinical Medical College of Lanzhou University; c Department of Radiology, The First Hospital of Lanzhou University; d Department of radiotherapy, Gansu Provincial People's Hospital, Lanzhou, China.

**Keywords:** breast cancer, diagnostic test accuracy, imaging diagnosis, network meta-analysis

## Abstract

**Background:**

: Breast cancer (BC) is the most common cancer in women all over the world and the second most common cause of cancer-related mortality. Imaging examination plays an important role in the diagnosis of early breast cancer. Due to different imaging principles and methods, all kinds of examinations have their advantages and disadvantages. It is particularly important for clinicians to choose these examination methods reasonably to achieve the best diagnostic effect. The objectives of this systematic review and NMA are to determine the diagnostic accuracy of imaging technologies for breast cancer and to compare the diagnostic accuracy of different index tests and to support guidelines development and clinical practice.

**Methods:**

: PubMed, Embase.com, the Cochrane Central Register of Controlled Trials (CENTRAL), Web of Science, China National Knowledge Infrastructure (CNKI), Wanfang, and SinoMed will be searched to identify relevant studies up to August 31, 2021. We will include random controlled trials, cross-sectional studies, case-control studies, and cohort studies that evaluate the diagnostic accuracy of different imaging diagnostic methods for breast cancer. The Quality Assessment of Diagnostic Accuracy Studies 2 quality assessment tool will be used to assess the risk of bias in each study. Standard pairwise meta-analysis and NMA will be performed using STATA V.12.0, MetaDiSc 1.40, and R 3.4.1 software to compare the diagnostic efficacy of different imaging diagnostic methods. Subgroup analyses and sensitivity analyses will be conducted to investigate the sources of heterogeneity.

**Results:**

: The results of this study will be published in a peer-reviewed journal.

**Conclusion:**

: This study will comprehensively evaluate the accuracy of different imaging diagnostic methods in the diagnosis of breast cancer. The results of this study will provide high-quality evidence to support clinical practice and guidelines development.

## 1 Introduction

Breast cancer (BC) is the most common cancer in women all over the world and the second most common cause of cancer-related mortality.^[1,2]^ There was no specific symptom in the early stage BC. The survival rate of breast cancer treatment is closely related to the stage of breast cancer.^[3–5]^ Relevant studies have shown that early detection and timely surgical treatment, the 5-year survival rate of patients is more than 80%. If it is advanced breast cancer, the prognosis is poor, the 5-year survival rate is less than 50%.^[6]^ How to make early diagnosis and predict prognosis, effectively guide the clinical and improve the treatment plan is one of the current clinical research directions. Improving the sensitivity and specificity of breast cancer diagnosis and eliminating false-positive cases have positive clinical significance for the early diagnosis of breast cancer and reducing its mortality.^[7–9]^ Imaging examination plays an important role in the diagnosis of early breast cancer. There are many methods for breast imaging. With the continuous development of medical technology, mammography, breast ultrasound, and MRI equipment continue to upgrade, MRI Dynamic enhancement, mammography tomography fusion technology has been widely used in the diagnosis of breast cancer.^[10,11]^ Due to different imaging principles and methods, all kinds of examinations have their advantages and disadvantages. It is particularly important for clinicians to choose these examination methods reasonably in order to achieve the best diagnostic effect.^[12,13]^ The objectives of this systematic review and NMA are to determine the diagnostic accuracy of imaging technologies for breast cancer and to compare the diagnostic accuracy of different index tests and to support guidelines development and clinical practice.

## 2 Methods

### 2.1 Design and registration

We will conduct an NMA of diagnostic test accuracy. The protocol of this study has been registered on the International Platform of Registered Systematic Review and Meta-Analysis Protocols (INPLASY, INPLASY202140041). We will follow the Preferred Reporting Items for Systematic Reviews and Meta-analysis of diagnostic test accuracy (PRISMA-DTA) statements for reporting our systematic review.^[14]^

### 2.2 Search strategy

We will search English databases: PubMed, Embase.com, the Cochrane Central Register of controlled trials (CENTRAL), and Web of Science, as well as Chinese databases: China National Knowledge Infrastructure (CNKI), Wanfang, and Sinomed. The keywords will include: Ultrasonography, X-Ray Microtomography, Echotomography, Ultrasonic Imaging, Medical Sonography, Ultrasonographic Imaging, Echography, Ultrasonic Diagnosis, MicroCT, X-Ray Micro-CAT Scan, X-Ray Micro-Computed Tomography, Xray MicroCT, sensitivity (SEN), specificity (SPE), false positive (FP) reactions, false negative (FN) reactions, ROC curve, breast cancer, breast tumor, breast cancer, breast cancer, breast tumor, breast cancer, and their synonym. Taking PubMed as an example, the specific retrieval strategy is shown in Table 1.

**Table 1:** Flow chart of literature screening.

#1	“Ultrasonography”[Mesh] OR Diagnostic Ultrasound∗[Title/Abstract] OR Ultrasound Imaging∗[Title/Abstract] OR Echotomography[Title/Abstract] OR Ultrasonic Imaging[Title/Abstract] OR Medical Sonography[Title/Abstract] OR Ultrasonographic Imaging∗[Title/Abstract] OR Echography[Title/Abstract] OR Ultrasonic Diagnosis∗[Title/Abstract] OR Computer Echotomography[Title/Abstract] OR Ultrasonic Tomography[Title/Abstract]
#2	“X-Ray Microtomography”[Mesh] OR “Tomography Scanners, X-Ray Computed”[Mesh] OR X Ray Microtomography[Title/Abstract] OR MicroCT∗[Title/Abstract] OR X-Ray Micro-CAT Scan∗[Title/Abstract] OR X-Ray Micro-Computed Tomography[Title/Abstract] OR Xray MicroCT∗[Title/Abstract] OR X-Ray Micro-CT Scan∗[Title/Abstract] OR X-Ray Microcomputed Tomography[Title/Abstract] OR X Ray Microcomputed Tomography[Title/Abstract] OR X-ray MicroCT∗[Title/Abstract] OR Xray Micro CT∗[Title/Abstract] OR Microcomputed Tomography[Title/Abstract]
#3	“Magnetic Resonance Imaging”[Mesh] OR NMR Imaging[Title/Abstract] OR MR Tomography[Title/Abstract] OR NMR Tomography[Title/Abstract] OR Steady State Free Precession MRI[Title/Abstract] OR Zeugmatography[Title/Abstract] OR Chemical Shift Imaging∗[Title/Abstract] OR Magnetic Resonance Image∗[Title/Abstract] OR Magnetization Transfer Contrast Imaging[Title/Abstract] OR MRI Scan∗[Title/Abstract] OR Proton Spin Tomography[Title/Abstract] OR fMRI[Title/Abstract] OR Functional MRI∗[Title/Abstract] OR Functional Magnetic Resonance Imaging[Title/Abstract] OR Spin Echo Imaging∗[Title/Abstract]
#4	#1 OR #2 OR #3
#5	“Sensitivity AND Specificity”[Mesh] OR “False Positive Reactions”[Mesh] OR “False Negative Reactions”[Mesh] OR “ROC Curve”[Mesh] OR “Predictive Value of Tests”[Mesh] OR sensitivity[Title/Abstract] OR specificity[Title/Abstract] OR receiver operating characteristic[Title/Abstract] OR receiver operator characteristic[Title/Abstract] OR predictive value∗[Title/Abstract] OR roc[Title/Abstract] OR pre-test odds[Title/Abstract] OR pretest odds[Title/Abstract] OR pre-test probability∗[Title/Abstract] OR pretest probability∗[Title/Abstract] OR post-test odds[Title/Abstract] OR posttest odds[Title/Abstract] OR post-test probabilit∗[Title/Abstract] OR posttest probabilit∗[Title/Abstract] OR likelihood ratio∗[Title/Abstract] OR positive predictive value∗[Title/Abstract] OR negative predictive value∗[Title/Abstract] OR false negative∗[Title/Abstract] OR false positive∗[Title/Abstract] OR true negative∗[Title/Abstract] OR true positive∗[Title/Abstract] OR fn[Title/Abstract] OR fp[Title/Abstract] OR tn[Title/Abstract] OR tp[Title/Abstract]
#6	“Breast Neoplasms”[Mesh] OR “Breast Carcinoma In Situ”[Mesh] OR “Breast Neoplasms, Male”[Mesh] OR “Carcinoma, Ductal, Breast”[Mesh] OR “Carcinoma, Lobular”[Mesh] OR “Inflammatory Breast Neoplasms”[Mesh] OR “Triple Negative Breast Neoplasms”[Mesh] OR “Unilateral Breast Neoplasms”[Mesh] OR breast neoplasm∗[Title/Abstract] OR breast tumor∗[Title/Abstract] OR breast carcinoma∗[Title/Abstract] OR breast cancer∗[Title/Abstract] OR breast tumour∗[Title/Abstract] OR mammary neoplasm∗[Title/Abstract] OR mammary tumor∗[Title/Abstract] OR mammary carcinoma∗[Title/Abstract] OR mammary cancer∗[Title/Abstract] OR mammary tumour∗[Title/Abstract] OR breast adenocarcinoma∗[Title/Abstract] OR breast carcinogenesis[Title/Abstract] OR breast sarcoma∗[Title/Abstract] OR phyllodes tumor∗[Title/Abstract] OR intraductal carcinoma∗[Title/Abstract] OR lobular carcinoma∗[Title/Abstract]
#7	#4 AND #5 AND #6

### 2.3 Inclusion and exclusion criteria

#### 2.3.1 Type of study

We will include random controlled trials, cross-sectional studies, case-control studies, and cohort studies that evaluated the diagnostic accuracy of different imaging methods for breast cancer. These may be either prospective or retrospective. There are no limitations in minimal quality, minimal sample size, or the number of patients. There will be no limitations on language, publication year, and publication status.

#### 2.3.2 Type of patients

Breast cancer patients over 18 years old confirmed by pathology or cytology have received 1 or more imaging methods including ultrasound examinations, molybdenum target X-ray, nuclear magnetic resonance, or combined examinations. There are no limitations in age, race, or nationality.

#### 2.3.3 Type of index tests

Breast cancer patients receive any kind of diagnostic ultrasound, molybdenum target X-ray examination, nuclear magnetic resonance examination, including B-ultrasound, contrast-enhanced ultrasound (CEUS), color Doppler ultrasound, full-field digital mammography (FFDM), contrast-enhanced spectral mammography (CESM), digital breast tomography (DBT), etc. It can be 1 or several imaging examinations.

#### 2.3.4 Reference standards

Pathology or cytology is the gold standard for the diagnosis of breast cancer.

#### 2.3.5 Type of outcomes

The primary outcomes are SEN, SPE, positive predictive value, negative predictive value, positive likelihood ratio (PLR), negative likelihood ratio (NLR), diagnostic odds ratio (DOR), area under the curve (AUC), and their respective 95% confidence interval.

#### 2.3.6 Exclusion criteria

1. Case report, literature review, case analysis, and review;
2. The original literature was deficient in experimental design;
3. The experimental design of the original literature is defective or not rigorous, including the inclusion and exclusion criteria are vague, the sample size is too small to demonstrate the argument, or the sample information is incomplete, and the statistical methods are not used properly.

### 2.4 Literature screening and data extraction

Two reviewers will independently screen the literature, extract the data, and cross-check the data. In case of disagreement, a third party will be consulted to assist in judgment, and the author will be contacted to supplement the missing data if possible. In the process of literature selection, we will first read the titles and abstracts. After excluding the unrelated literatures, we will further read the full text to determine whether they are included. A draft data extraction sheet will be developed using Microsoft Excel 2013 (Microsoft Corp, Redmond, WA, www.microsoft.com). Data extraction will include: author name, year of publication, country of the first author, number of authors, journal name, country of journals, funding, types of studies, age and number of participants, number and name of imaging examination, number and name of reference test, the reported number of TPs, FNs, TNs, and FPs. If studies did not report these values, we will attempt to reconstruct the 2 × 2 tables from the diagnostic estimates presented in the article for each imaging examination.

### 2.5 Assessment of risk of bias in included studies

Two review authors will independently assess the risk of bias in each study according to predefined criteria. We will resolve any disagreement by discussion or by involving a third assessor. The Quality Assessment of Diagnostic Accuracy Studies 2 quality assessment tool (QUADAS-2) will be used to assess the methodological quality.^[15]^ QUADAS-2 is composed of 4 important parts:

1. case selection;
2. to be evaluated diagnosis test;
3. diagnostic gold standard;
4. case selection process and progress.

Two independent evaluators will answer and evaluate each part of the questions one by one, and negotiate if they are inconsistent solve. The evaluation results will be recorded in the form of QUADAS-2.^[16]^

### 2.6 Geometry of the network

A network plot will be drawn to describe and present the geometry of index tests using R software V.3.4.1. Trials will be excluded if they are not connected by index tests. Nodes in network geometry represent different imaging methods and edges represent head-to-head comparisons. The size of nodes and thickness of edges are associated with sample sizes of index tests and numbers of included trials, respectively.

### 2.7 Network meta-analysis

#### 2.7.1 Pairwise meta-analyses

We will use STATA V.12.0 (Stata) and MetaDiSc 1.40 for constructing forest plots showing estimates of SEN, SPE, PLR, NLR, DOR, and their corresponding 95% confidence intervals for each imaging method. Chi^2^ test will be used to analyze the statistical heterogeneity of the results, and *P* value and *I*^2^ will be used to quantitatively judge the heterogeneity. If the homogeneity of the included studies is low (*P* > .1 and *I*^2^ < 50%), the fixed-effect model will be used for meta-analysis; if there is heterogeneity between the included studies (*P* < .1 and *I*^2^ ≥ 50%), the source of heterogeneity will be further analyzed. After excluding the influence of obvious clinical heterogeneity, the random effect model will be used for meta-analysis. We will draw the summary receiver operating characteristic curve. The area under the curve (AUC) will be calculated. The larger the AUC is, the closer it is to 1, which indicates that the authenticity of the diagnosis using this method is better. In addition, we will use STATA V.12.0 (Stata) and Review Manager 5.30 (RevMan) analysis software to build the hierarchical summary receiver operating characteristic curves graphics for each imaging method.^[17]^

#### 2.7.2 Indirect comparisons between competing diagnostic tests

We will calculate relative diagnostic outcomes between each imaging method including relative SEN, relative SPE, relative DOR, relative PLR, and relative NLR.^[18]^ Then, we will conduct indirect comparisons using the relative diagnostic outcomes. All analysis will be performed using STATA V.12.0 (Stata) software.

### 2.8 Publication bias

The publication bias will be explored using the Deek test for outcomes with studies no less than 10.^[19]^

### 2.9 Subgroup analysis and meta-regression analysis

If sufficient studies are available, subgroup analysis or univariate meta-regression analysis will be performed on the within-study factors (time, sample size) and between study factors (mean age, race) respectively to screen out the important factors leading to heterogeneity.

## 3 Result

### 3.1 Screening results

**Figure 1:**
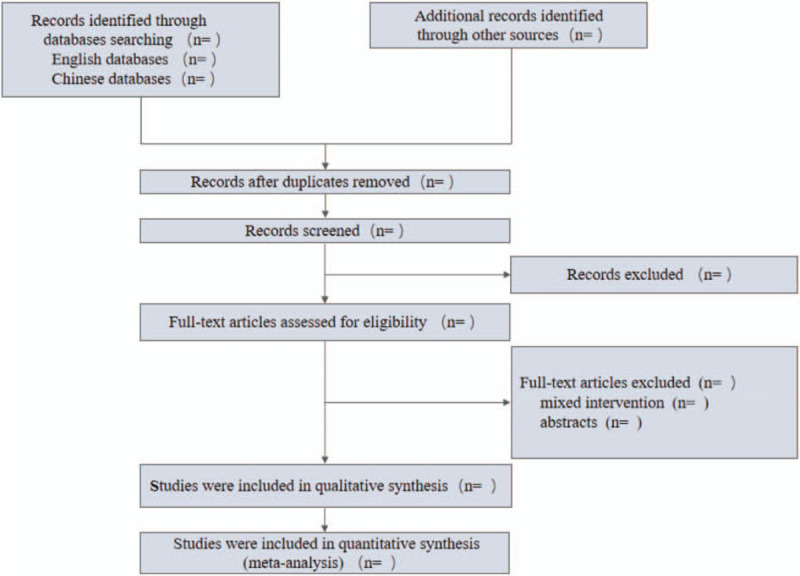
Flow chart of literature screening.

Two reviewers will perform the titles, abstracts, and full-texts screening, and we will present the screening process in a PRISMA flow plot (Fig. 1).

### 3.2 General characteristics and quality of studies

We presented characteristics of some included studies in Table 2. The gold standard for all studies was pathology. The details are shown in Table 2.

**Table 2:** Characteristics of partially included studies.

							Ultrasonic				Molybdenum target				MRI				
First author	Year	Country	Language	Method	Age	Total number of lesions	TP	FP	FN	TN	TP	FP	FN	TN	TP	FP	FN	TN	Gold standard
Tamerozuikel^[20]^	2010	Turkey	English	prospective	average 46.1	46	11	4	5	26	13	11	3	19	13	8	3	22	Pathological examination
Federica^[21]^	2009	Rome	English	retrospective	average 45.7	97	47	25	8	17	40	23	15	19	54	2	1	40	Pathological examination
Zhang Yongting^[22]^	2019	China	Chinese	retrospective	27-63	50	13	18	9	10	20	5	12	13	36	7	5	2	Pathological examination
Liu Xiaowei^[23]^	2019	China	Chinese	retrospective	38-46	65	41	4	8	12	39	5	10	11	32	6	17	10	Pathological examination
Guo Xiaoliang^[24]^	2020	China	Chinese	retrospective	44.3 ± 5.2	167	66	20	10	71	68	26	8	65	70	16	6	75	Pathological examination
Xia Xiaotian^[25]^	2010	China	Chinese	retrospective	average 54	117	60	9	8	40	55	6	13	43	66	10	2	39	Pathological examination

## 4 Discussion

Improving the sensitivity and specificity of breast cancer diagnosis and eliminating false-positive cases have positive clinical significance for the early diagnosis of breast cancer and reducing its mortality. This NMA will summarize the direct and indirect evidence to assess the diagnostic accuracy of different imaging methods for breast cancer and attempt to find the most effective imaging method for the diagnosis of breast cancer. We hope to help clinicians make more accurate diagnosis decisions.

## Author contributions

**Conceptualization:** Mei Zhang, Rongna Lian, Ruinian Zhang, Yulong Hong, Wen Feng, Shifang Feng.

**Funding acquisition:** Shifang Feng.

**Methodology:** Mei Zhang, Rongna Lian, Ruinian Zhang, Wen Feng, Shifang Feng.

**Software:** Mei Zhang, Rongna Lian, Ruinian Zhang, Yulong Hong, Shifang Feng.

**Writing – original draft:** Mei Zhang.
